# Supplementary material for: Identification and characterization of a novel heparan sulfate-binding domain in Activin A longest variants and implications for function
Source: PLoS One. 2019 Sep 19;14(9):e0222784. doi: 10.1371/journal.pone.0222784 (PMC6752817; doi:10.1371/journal.pone.0222784)
Supplement: S1 Fig — Each Activin has 2 exons. For each Activin, the top diagram delineates Genomic organization and the lower diagram, spliced mRNA. The chromosomal location and sequence homology of Activins C and E suggest they arose from genome duplication. Exon organization was determined using the BLAT search engine, UCSC Genome Browser (genome.ucsc.edu) and are not drawn to scale. (DOCX) [file pone.0222784.s001.docx]

**Figure S1.** Genomic organization of human Activin B, C & E. Each Activin has 2 exons. For each Activin, the top diagram delineates Genomic organization and the lower diagram, spliced mRNA. The chromosomal location and sequence homology of Activins C and E suggest they arose from genome duplication. Exon organization was determined using the BLAT search engine, UCSC Genome Browser (genome.ucsc.edu) and are not drawn to scale.

5’

3’

5’

3’

5’

3’

INHBB: Ch2p14.2

INHBC: Ch12p13.3

INHBE: Ch12p13.3

5’

3’

5’

3’

5’

3’
