# Supplementary material for: Identification and characterization of a novel heparan sulfate-binding domain in Activin A longest variants and implications for function
Source: PLoS One. 2019 Sep 19;14(9):e0222784. doi: 10.1371/journal.pone.0222784 (PMC6752817; doi:10.1371/journal.pone.0222784)
Supplement: S2 Fig — A. Schematic representation of Activin proteins. Each protein consists of an N-terminal signal peptide (SP), a prodomain and mature ligand. Red Arrow: furin cleavage site. B. Protein sequences of human Act A, B, C and E are listed. Exons are shaded in different colors. Underlined sequences are putative heparin binding domains (Act AX178-102 and Act AX3259-272). Residues highlighted in Red have codons shared by adjoining exons. Numbers in parenthesis denote position of the processed, active ligand (Bold type). (DOCX) [file pone.0222784.s002.docx]

**Figure S2**. Protein Sequences of full-length human Activins. A. Schematic representation of Activin proteins. Each protein consists of an N-terminal signal peptide (SP), a prodomain and mature ligand. Red Arrow: furin cleavage site. B. Protein sequences of human Act A, B, C and E are listed. Exons are shaded in different colors. Underlined sequences are putative heparin binding domains (Act AX^178-102^ and Act AX3^259-272^). Residues highlighted in Red have codons shared by adjoining exons. Numbers in parenthesis denote position of the processed, active ligand (**Bold** type).

**A.**


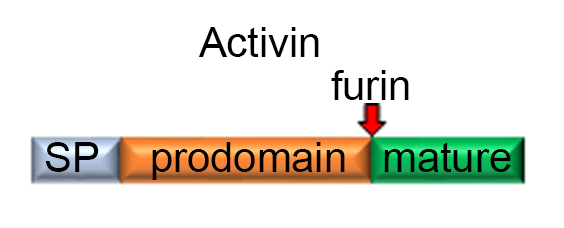


**B.**

INHBA X1 (433-548) XP_016867663

>XP_016867663 inhibin beta A chain isoform X1 [Homo sapiens] MPTLNRDSCCHSCQKGGKNQELLLKEVALAGAQGKNRGCRLDRLGPGSRHPASWARAAAAFRRDPWKLPAGAAQENLL**KRRRKKKTPKKKIKKSTHTKKPAR**EGGGKAGPFKKAITTTFAARMPLLWLRGFLLASCWIIVRSSPTPGSEGHSAAPDCPSCALAALPKDVPNSQPEMVEAVKKHILNMLHLKKRPDVTQPVPKAALLNAIRKLHVGKVGENGYVEIEDDIGRRAEMNELMEQTSEIITFAESGTARKTLHFEISKEGSDLSVVERAEVWLFLKVPKANRTRTKVTIRLFQQQKHPQGSLDTGEEAEEVGLKGERSELLLSEKVVDARKSTWHVFPVSSSIQRLLDQGKSSLDVRIACEQCQESGASLVLLG**KKKKKEEEGEGKKK**GGGEGGAGADEEKEQSHRPFLMLQARQSEDHPHRRRRR**GLECDGKVNICCKKQFFVSFKDIGWNDWIIAPSGYHANYCEGECPSHIAGTSGSSLSFHSTVINHYRMRGHSPFANLKSCCVPTKLRPMSMLYYDDGQNIIKKDIQNMIVEECGCS**

INHBA X2 (368-483) XP_016867664

>XP_016867664 inhibin beta A chain isoform X2 [Homo sapiens]

MELITQGDPENLLKRRRKKKTPKKKIKKSTHTKKPAREGGGKAGPFKKAITTTFAARMPLLWLRGFLLASCWIIVRSSPTPGSEGHSAAPDCPSCALAALPKDVPNSQPEMVEAVKKHILNMLHLKKRPDVTQPVPKAALLNAIRKLHVGKVGENGYVEIEDDIGRRAEMNELMEQTSEIITFAESGTARKTLHFEISKEGSDLSVVERAEVWLFLKVPKANRTRTKVTIRLFQQQKHPQGSLDTGEEAEEVGLKGERSELLLSEKVVDARKSTWHVFPVSSSIQRLLDQGKSSLDVRIACEQCQESGASLVLLGKKKKKEEEGEGKKKGGGEGGAGADEEKEQSHRPFLMLQARQSEDHPHRRRRR**GLECDGKVNICCKKQFFVSFKDIGWNDWIIAPSGYHANYCEGECPSHIAGTSGSSLSFHSTVINHYRMRGHSPFANLKSCCVPTKLRPMSMLYYDDGQNIIKKDIQNMIVEECGCS**

INHBA X3 (311-426) NP_002183

> NP_002183 inhibin beta A chain isoform X3 [Homo sapiens]

MPLLWLRGFLLASCWIIVRSSPTPGSEGHSAAPDCPSCALAALPKDVPNSQPEMVEAVKKHILNMLHLKKRPDVTQPVPKAALLNAIRKLHVGKVGENGYVEIEDDIGRRAEMNELMEQTSEIITFAESGTARKTLHFEISKEGSDLSVVERAEVWLFLKVPKANRTRTKVTIRLFQQQKHPQGSLDTGEEAEEVGLKGERSELLLSEKVVDARKSTWHVFPVSSSIQRLLDQGKSSLDVRIACEQCQESGASLVLLG**KKKKKEEEGEGKKK**GGGEGGAGADEEKEQSHRPFLMLQARQSEDHPHRRRRR**GLECDGKVNICCKKQFFVSFKDIGWNDWIIAPSGYHANYCEGECPSHIAGTSGSSLSFHSTVINHYRMRGHSPFANLKSCCVPTKLRPMSMLYYDDGQNIIKKDIQNMIVEECGCS**

INHBB (293-407) NP_002184

>NP_002184 inhibin beta B chain preproprotein [Homo sapiens]

MDGLPGRALGAACLLLLAAGWLGPEAWGSPTPPPTPAAPPPPPPPGSPGGSQDTCTSCGGFRRPEELGRVDGDFLEAVKRHILSRLQMRGRPNITHAVPKAAMVTALRKLHAGKVREDGRVEIPHLDGHASPGADGQERVSEIISFAETDGLASSRVRLYFFISNEGNQNLFVVQASLWLYLKLLPYVLE**KGSRRKVRVK**VYFQEQGHGDRWNMVEKRVDLKRSGWHTFPLTEAIQALFERGERRLNLDVQCDSCQELAVVPVFVDPGEESHRPFVVVQARLGDSRHRIRKR**GLECDGRTNLCCRQQFFIDFRLIGWNDWIIAPTGYYGNYCEGSCPAYLAGVPGSASSFHTAVVNQYRMRGLNPGTVNSCCIPTKLSTMSMLYFDDEYNIVKRDVPNMIVEECGCA**

INHBC (237-352) NP_005529

>NP_005529 inhibin beta C chain preproprotein [Homo sapiens]

MTSSLLLAFLLLAPTTVATPRAGGQCPACGGPTLELESQRELLLDLAKRSILDKLHLTQRPTLNRPVSRAALRTALQHLHGVPQGALLEDNREQECEIISFAETGLSTINQTRLDFHFSSDRTAGDREVQQASLMFFVQLPSNTTWTLKVRVLVLGPHNTNLTLATQYLLEVDASGWHQLPLGPEAQAACSQGHLTLELVLEGQVAQSSVILGGAAHRPFVAARVRVGGKHQIHRR**GIDCQGGSRMCCRQEFFVDFREIGWHDWIIQPEGYAMNFCIGQCPLHIAGMPGIAASFHTAVLNLLKANTAAGTTGGGSCCVPTARRPLSLLYYDRDSNIVKTDIPDMVVEACGCS**

INHBE (237-350) NP_113667

NP_113667 inhibin beta E chain preproprotein [Homo sapiens]

MRLPDVQLWLVLLWALVRAQGTGSVCPSCGGSKLAPQAERALVLELAKQQILDGLHLTSRPRITHPPPQAALTRALRRLQPGSVAPGNGEEVISFATVTDSTSAYSSLLTFHLSTPRSHHLYHARLWLHVLPTLPGTLCLRIFRWGPRRRRQGSRTLLAEHHITNLGWHTLTLPSSGLRGEKSGVLKLQLDCRPLEGNSTVTGQPRRLLDTAGHQQPFLELKIRANEPGAGRARRR**TPTCEPATPLCCRRDHYVDFQELGWRDWILQPEGYQLNYCSGQCPPHLAGSPGIAASFHSAVFSLLKANNPWPASTSCCVPTARRPLSLLYLDHNGNVVKTDVPDMVVEACGCS**
