# Supplementary material for: Identification and characterization of a novel heparan sulfate-binding domain in Activin A longest variants and implications for function
Source: PLoS One. 2019 Sep 19;14(9):e0222784. doi: 10.1371/journal.pone.0222784 (PMC6752817; doi:10.1371/journal.pone.0222784)
Supplement: S3 Fig — Proteolytic processing is catalyzed by a proprotein convertase/furin protease between a conserved cluster of basic residues (Arg) and hydrophobic/neutral residue (Gly or Thr; red arrow). (DOCX) [file pone.0222784.s003.docx]

**Figure S3** Border region between prodomain (light gray) and active ligand (dark gray) of Activins. Proteolytic processing is catalyzed by a proprotein convertase/furin protease between a conserved cluster of basic residues (Arg) and hydrophobic/neutral residue (Gly or Thr; red arrow).

INHBA   QARQSEDHPHRRRRRGLECDGKVNICC
INHBB   QARLGDSR-HRIRKRGLECDGRTNLCC
INHBC   -ARVRVGGKHQIHRRGIDCQGGSRMCC
INHBE   -IRANEPGAGRARRRTPTCEPATPLCC
