# Supplementary material for: Identification and characterization of a novel heparan sulfate-binding domain in Activin A longest variants and implications for function
Source: PLoS One. 2019 Sep 19;14(9):e0222784. doi: 10.1371/journal.pone.0222784 (PMC6752817; doi:10.1371/journal.pone.0222784)
Supplement: S4 Fig — A. Amino acid alignments were generated with T-Coffee alignment tool (www.tcoffee.org) and UGENE Integrated Bioinformatics Tools (ugene.net). B. Models were generated by the I-TASSER server for protein structure and function prediction [Yang, et al. (2015) The I-TASSER Suite: protein structure and function prediction. Nature methods 12, 7–8] and are derived from PDB: 2ARV. N-terminus is in red. (DOCX) [file pone.0222784.s004.docx]

**Figure S4** Alignment and structure of human Activin ligands. A. Amino acid alignments were generated with T-Coffee alignment tool ([www.tcoffee.org](http://www.tcoffee.org)) and UGENE Integrated Bioinformatics Tools (ugene.net). B. Models were generated by the I-TASSER server for protein structure and function prediction [Yang, et al. (2015) The I-TASSER Suite: protein structure and function prediction. Nature methods **12**, 7-8] and are derived from PDB: 2ARV. N-terminus is in red.

**A.**


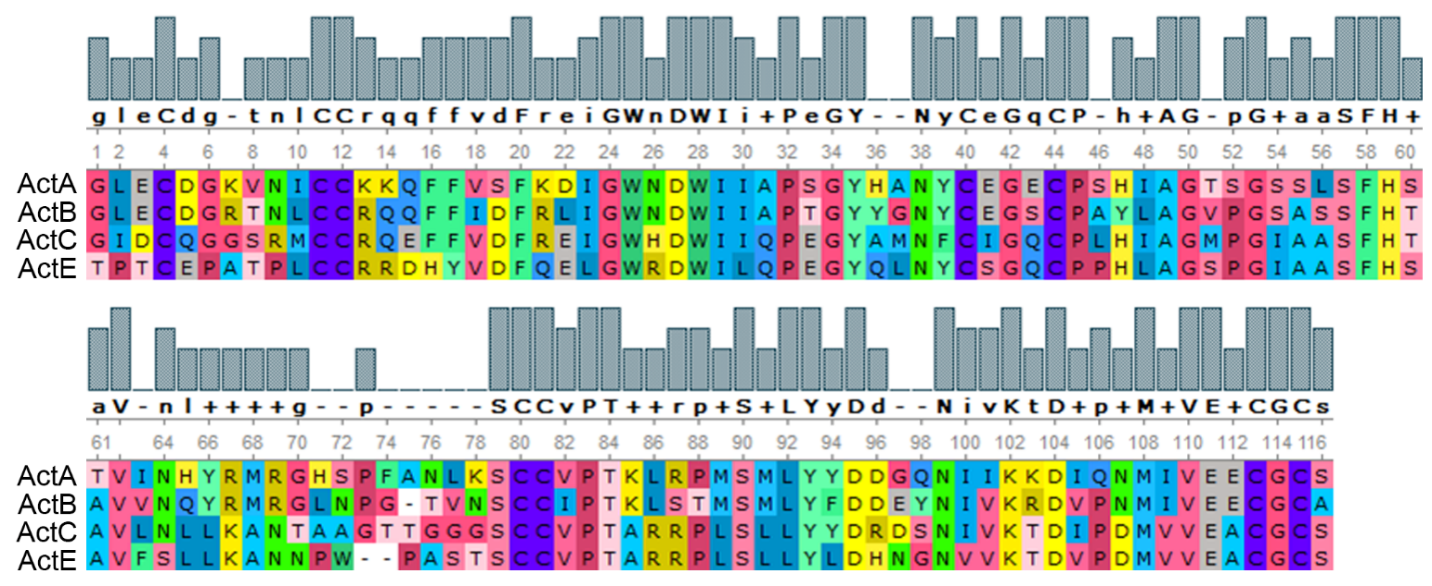


**B.
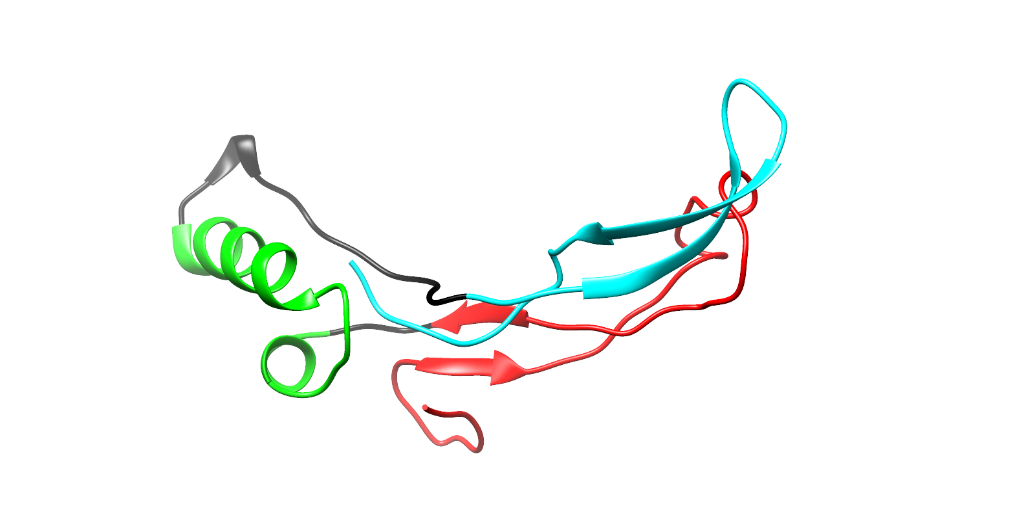

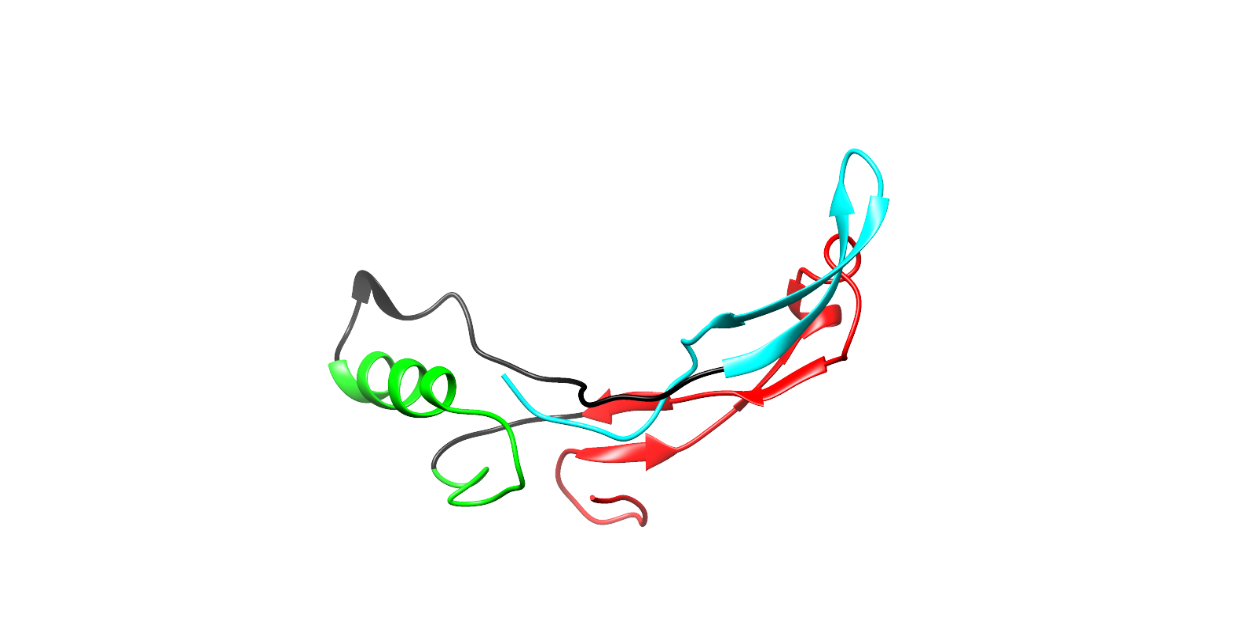

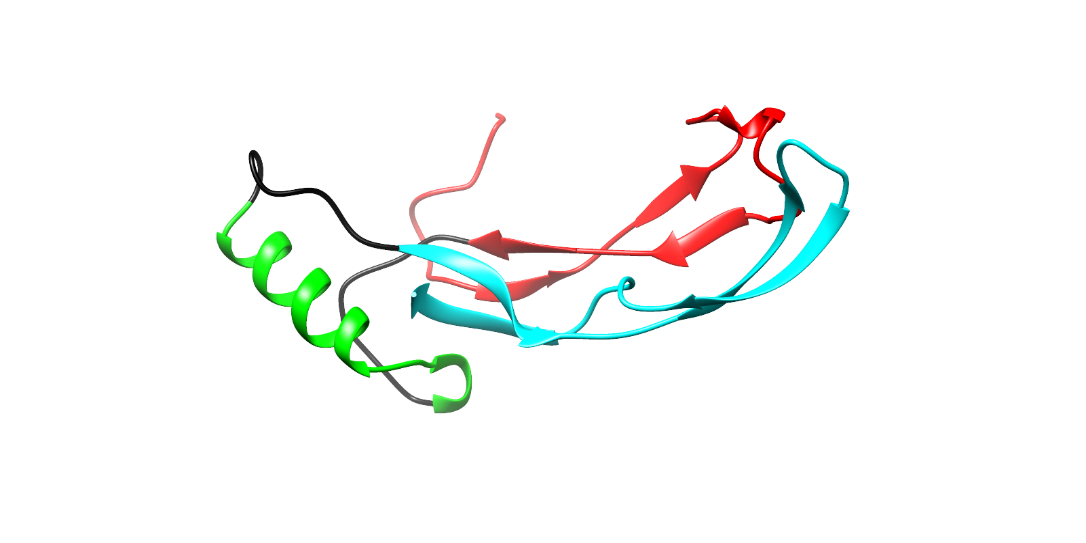

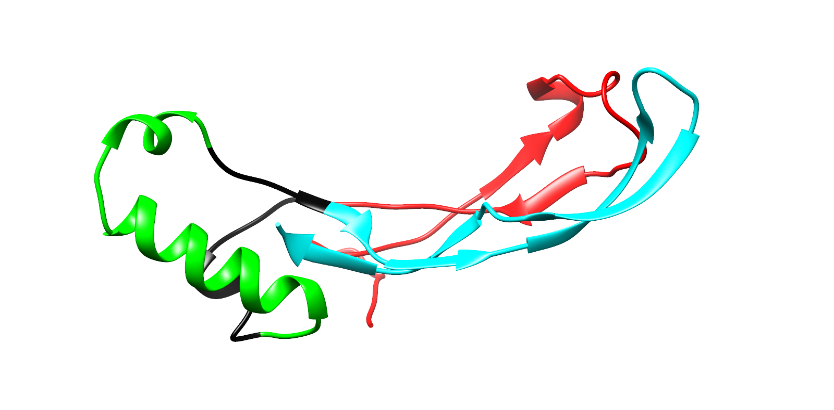
**

ActA

ActB

ActE

ActC
