# Supplementary material for: Identification and characterization of a novel heparan sulfate-binding domain in Activin A longest variants and implications for function
Source: PLoS One. 2019 Sep 19;14(9):e0222784. doi: 10.1371/journal.pone.0222784 (PMC6752817; doi:10.1371/journal.pone.0222784)
Supplement: S5 Fig — Activin transcripts were amplified from cDNAs prepared from human cells and tissues, using PCR primers listed in Table 4 in the manuscript. PCR reactions were run on 1% agarose gels and products were purified and subjected to Sanger Sequencing. The sequencing results were Blasted (blast.ncbi.nlm.nih.gov/Blast.cgi) to confirm identity. Screen shots of Nucleotide Blast results for human Act A, B, C and E are included. Diagrams below each screen shot show the target sequence and primers used for sequencing (Table 4). Also see Fig 1 in the manuscript and S1 Fig. (DOCX) [file pone.0222784.s005.docx]

**Figure S5** Sanger Sequencing Results. Activin transcripts were amplified from cDNAs prepared from human cells and tissues, using PCR primers listed in Table 4 in the manuscript. PCR reactions were run on 1% agarose gels and products were purified and subjected to Sanger Sequencing. The sequencing results were Blasted (blast.ncbi.nlm.nih.gov/Blast.cgi) to confirm identity. Screen shots of Nucleotide Blast results for human Act A, B, C and E are included. Diagrams below each screen shot show the target sequence and primers used for sequencing (Table 4). Also see Fig 1 in the manuscript and Fig. S1.

**
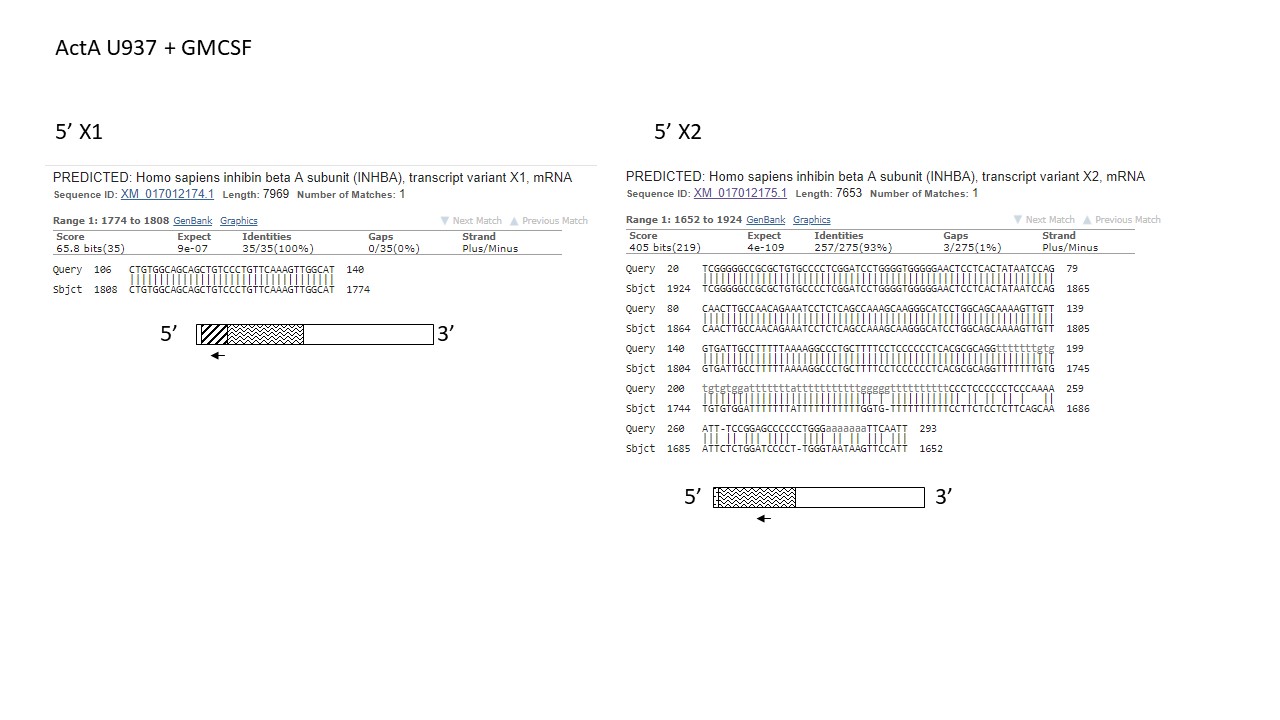

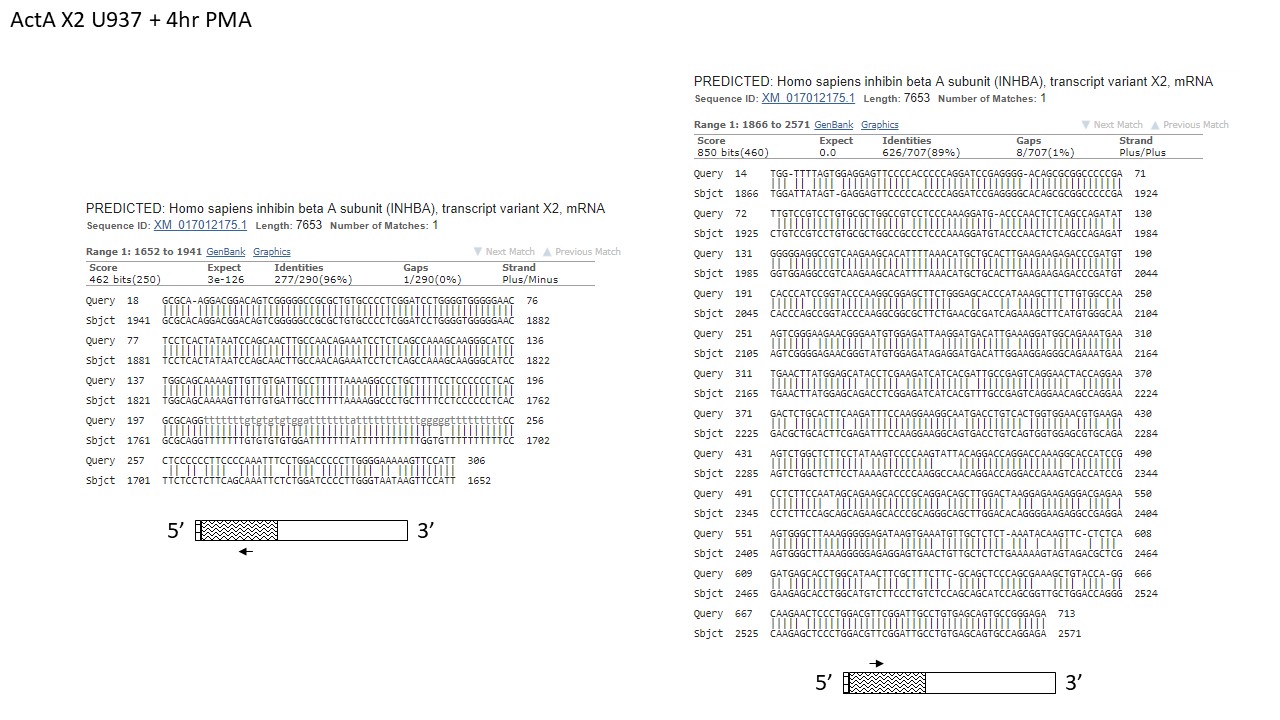
**

**
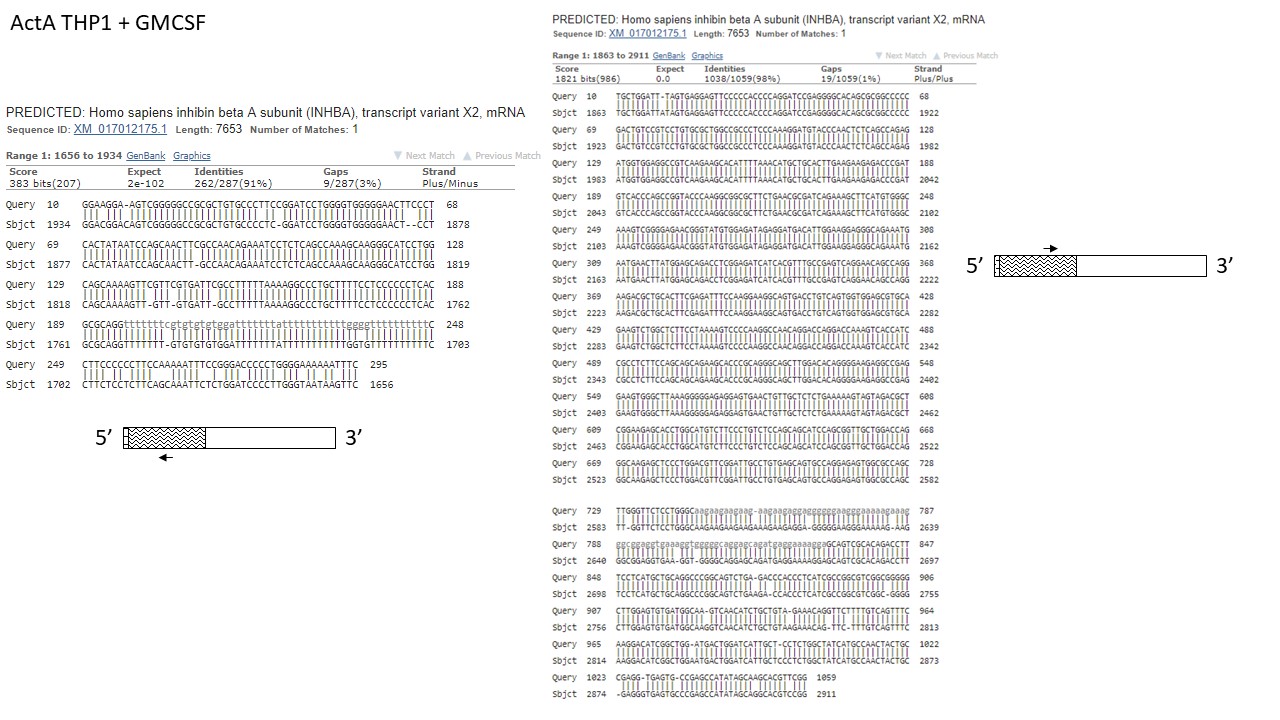
**

**
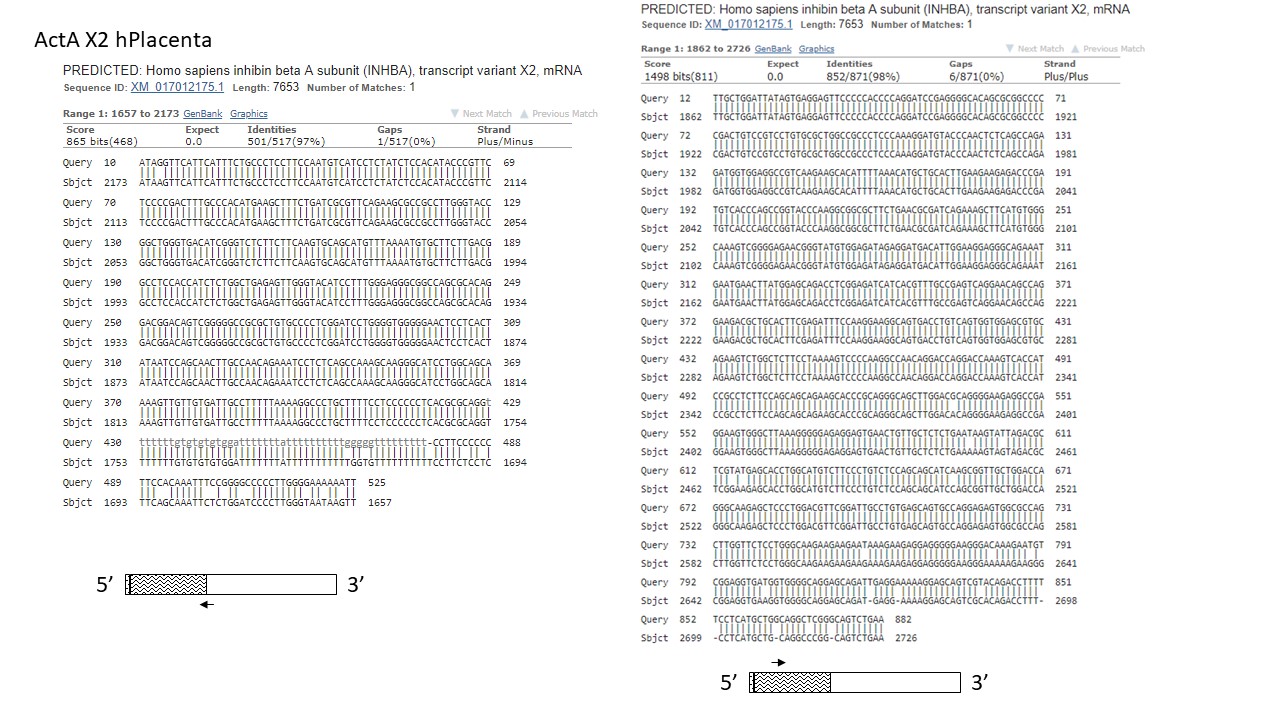
**

**
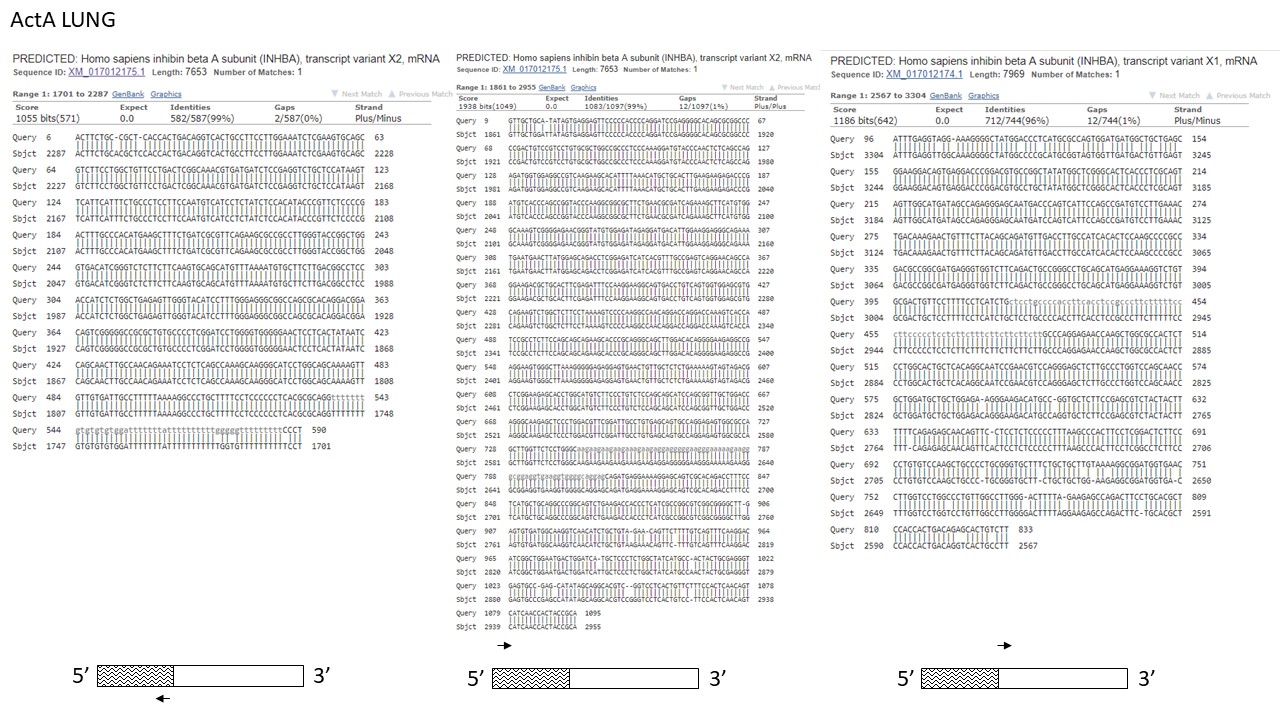
**

**
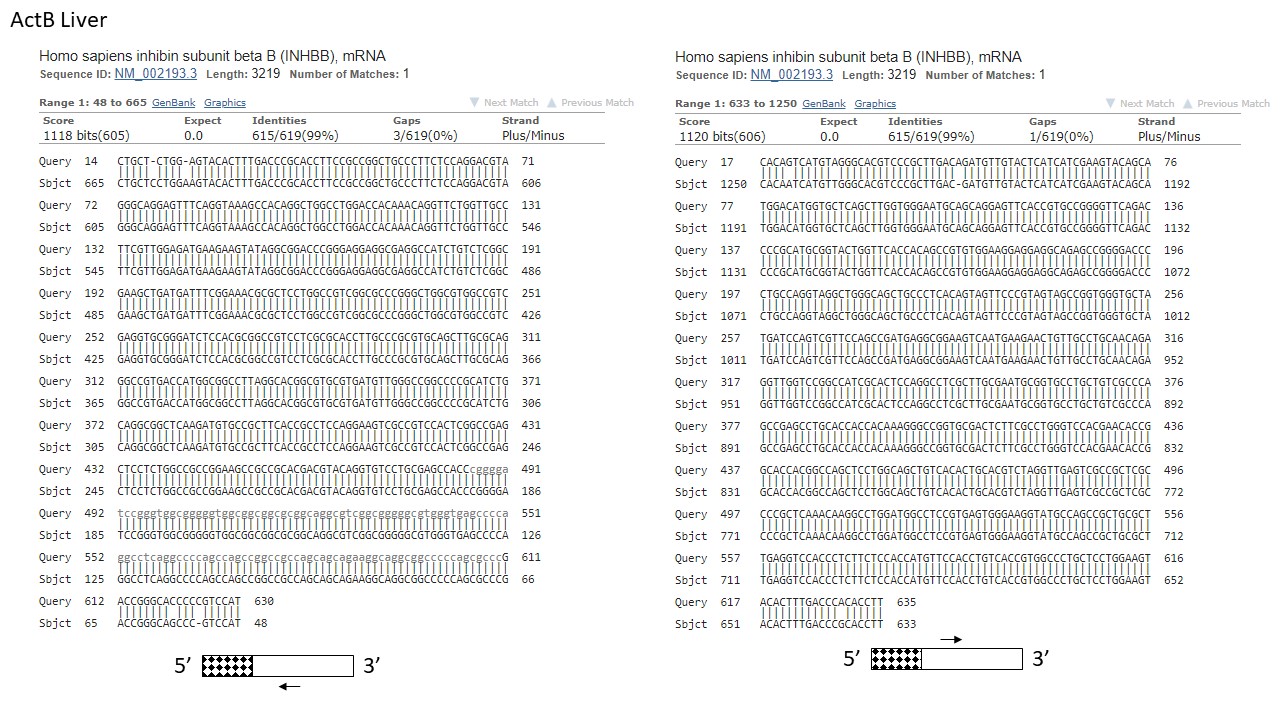
**

**
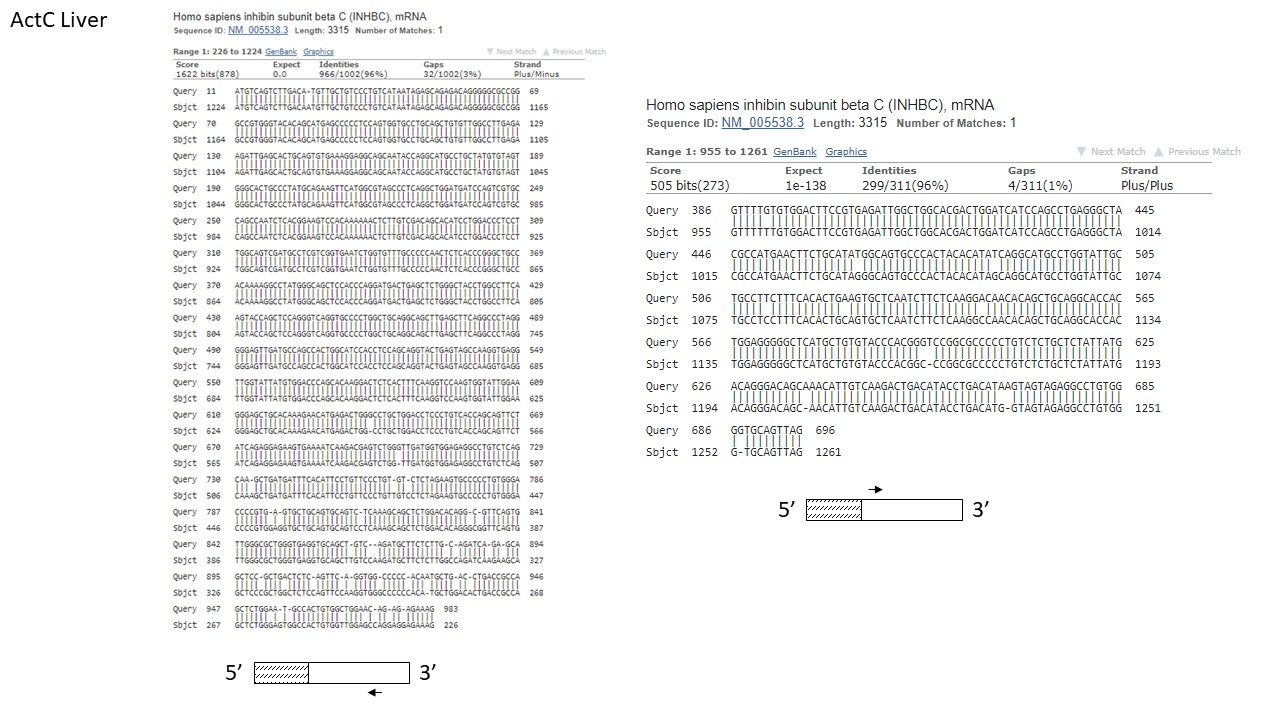
**

**
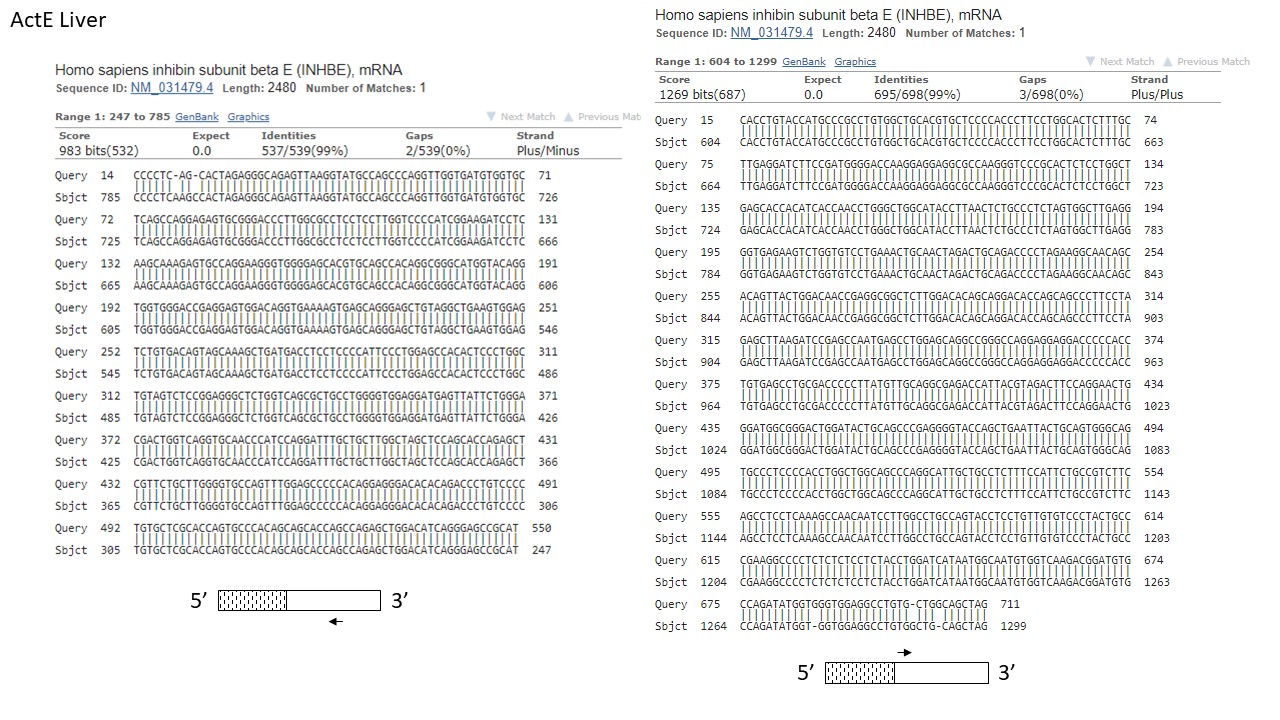
**
