# Supplementary material for: Identification and characterization of a novel heparan sulfate-binding domain in Activin A longest variants and implications for function
Source: PLoS One. 2019 Sep 19;14(9):e0222784. doi: 10.1371/journal.pone.0222784 (PMC6752817; doi:10.1371/journal.pone.0222784)

Activin A expression in AD293 cells; Uncroped gels (Fig. 4)

ActA variants (AD293) TALON column purified Anti-HIS Ab

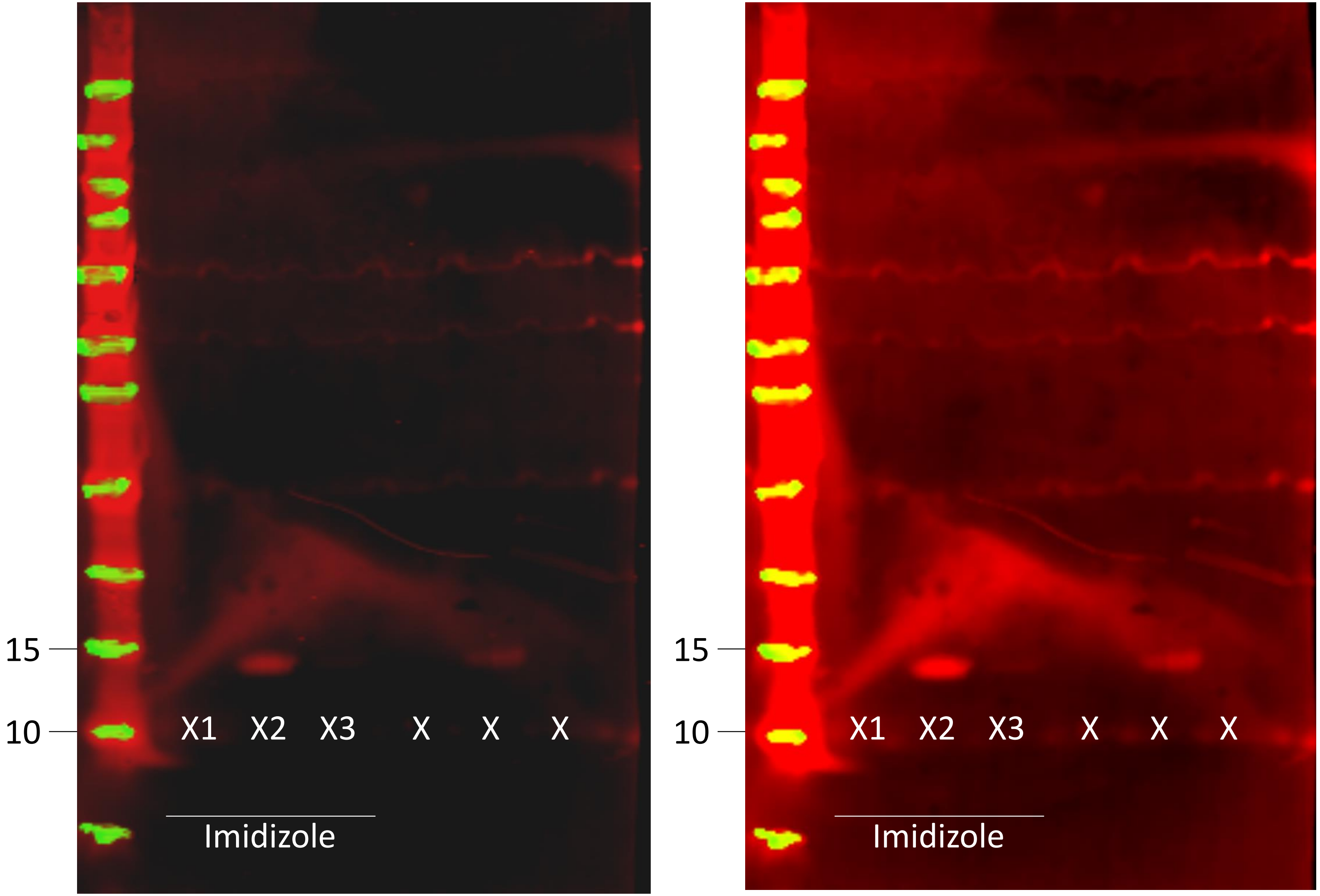

Mass markers: Novex Sharp Pre-Stained Protein Standard.

ActA variants (Cos1) TALON column purified Anti-HIS Ab (Fig. 4)

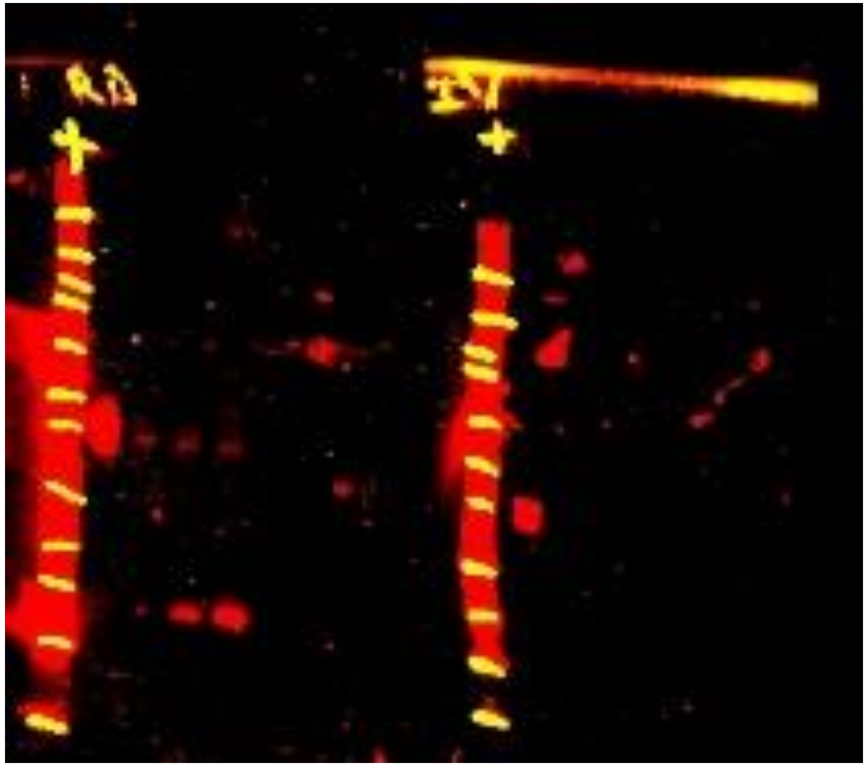
x x x x

Mass markers: Novex Sharp Pre-Stained Protein Standard.

ActA Pro-regions (AD293) TALON column purified Anti-HIS Ab (Fig. 8)

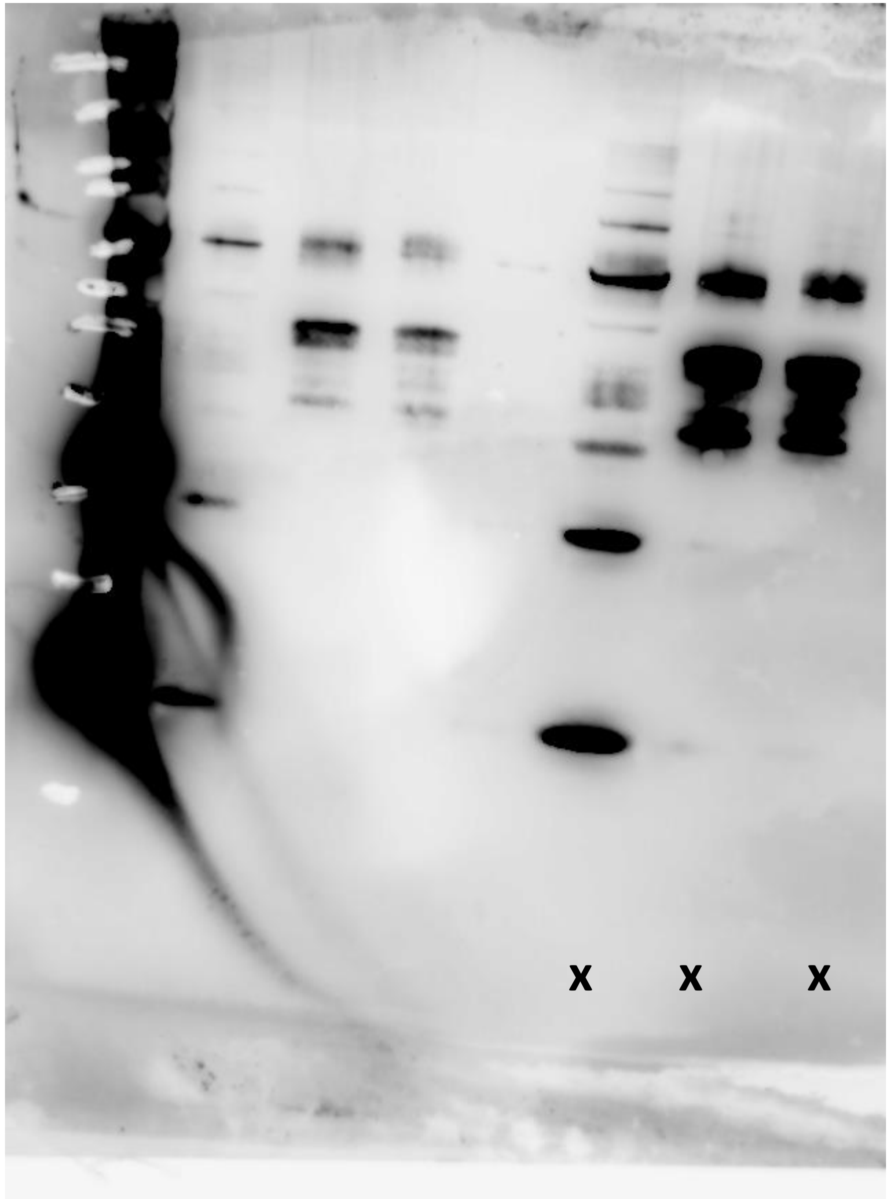

Supplement: S1 Raw Images — (PDF) [file pone.0222784.s008.pdf]
