# Supplementary material for: Identification and characterization of a novel heparan sulfate-binding domain in Activin A longest variants and implications for function
Source: PLoS One. 2019 Sep 19;14(9):e0222784. doi: 10.1371/journal.pone.0222784 (PMC6752817; doi:10.1371/journal.pone.0222784)
Supplement: Attachment — Submitted filename: Coverletter-PB-PLOS_8.27.19.docx [file pone.0222784.s009.docx]

**Translational Research Program in Pediatric Orthopaedics**

**Children’s Hospital of Philadelphia**

904 Abramson Research Center

3615 Civic Center Boulevard

Philadelphia, PA 19104

Aug 27, 2019

RE: PONE-D-19-18061; Identification and characterization of a novel heparan sulfate-binding domain in Activin A longest variants and implications for function

Nikos K Karamanos, Ph.D.

Academic Editor

PLOS ONE

Dear Dr. Karamanos:

Enclosed, please find a revised version of our above referenced manuscript (PONE-D-19-18061). We thank you and the Reviewers for the favorable evaluation of our work and giving us the opportunity to submit a revised manuscript. Below is our response to each comment and question raised by the Editor and Reviewers. Changes to the original version are indicated in **Bold** type.

**Editorial comments**

1. When submitting your revision, we need you to address these additional requirements.

Please ensure that your manuscript meets PLOS ONE's style requirements, including those for file naming. The PLOS ONE style templates can be found at

http://www.journals.plos.org/plosone/s/file?id=wjVg/PLOSOne_formatting_sample_main_body.pdf and http://www.journals.plos.org/plosone/s/file?id=ba62/PLOSOne_formatting_sample_title_authors_affiliations.pdf

**Response**

As requested, we have modified out manuscript to conform to PLOS ONE's style requirements and file naming.

2. Please include captions for your Supporting Information files at the end of your manuscript, and update any in-text citations to match accordingly. Please see our Supporting Information guidelines for more information: http://journals.plos.org/plosone/s/supporting-information.

**Response**

As requested, we have included captions for our Supporting Information files at the end of our manuscript, and updated any in-text citations to match accordingly.

3. Regarding blot/gel data: PLOS ONE now requires that submissions reporting blots or gels include original, uncropped blot/gel image data as a supplement or in a public repository. This is in addition to complying with our image preparation guidelines described at https://journals.plos.org/plosone/s/figures#loc-blot-and-gel-reporting-requirements. These requirements apply both to the main figures and to cropped blot/gel images included in Supporting Information. If the manuscript is positively reviewed, we will ask the authors to provide any missing raw image data for blot/gel results when they submit their first revision. As part of your review, please ensure that figures reporting blot or gel images comply with the journal’s image preparation guidelines and that the original data are provided following the journal’s request.

**Response**

As requested, we have included uncropped blot/gel images.

**Reviewers Comments**

Reviewer #1: In this work, authors studied the capacity of the variants X1 and X2 of Activin A to bind heparan sulfate. The experimental procedures included the analysis of human placental RNA and the exposure of human monocytic-like cells to phorbol ester and GM-CSF that stimulates the differentiation to macropohages, where there was noted an increased RNA synthesis of X1 and X2. Synthesis and secretion of all the three variants were tested by transfecting AD293 and COS1 cells, where X2 and X3 were detected. Authors were then showed the binding characteristics of the variants and 3D-structure and protein conformation.

Generally, this manuscript is very well written, clear and well presented. Experiments contain controls and the discussion of the results presents the contribution of this work in the to-date literature.

**Response**

We thank Reviewer 1 for their favorable and encouraging assessment of our work.

Reviewer #2: This manuscript is a relevant contribution in Act A studies. Authors successfully showed the expression and biological activity of X1 and X2 variants. Finally, they presented data regarding X1 and X2 interactions with heparan sulfate, describing a possible regulation mechanism of Act A-related processes. The manuscript is clear and well written and, in order to further improve the quality of the paper, please find bellow some minor suggestions.

Abstract - Please specify in what context the other variants are expressed or change the following sentence adequately - “Here, we asked whether the other variants are expressed and what additional structural features may be provided by their longer pro-regions”.

**Response**

As suggested, we have revised our original statement, which now reads: Here, we asked whether these other variants are expressed by human cells in vitro and what additional structural features are contained within their longer pro-regions.

Discussion - I suggest including a topic in discussion associating Act A interactions with HS regarding HS’ sulfation pattern.

**Response**

As suggested by the Reviewer, we have added a new paragraph to the Discussion section of the revised manuscript.

We hope the revisions and modifications made to our manuscript fully address the Reviewers’ comments and questions and make our study acceptable for publication. We would like to thank once again the Editor and Reviewers’ for their careful evaluation and constructive criticism of our work.

Sincerely,

Paul Billings, Ph. D.

The Children's Hospital of Philadelphia

Division of Orthopaedic Surgery

Philadelphia, PA 19104

Ph: 267-426-9691/425-2077

Email: BillingsP@email.chop.edu
